# Healing Efficacy of *Glycyrrhiza glabra* Extract Hydrogels in Experimental Second-Degree Burns

**DOI:** 10.3390/gels11100834

**Published:** 2025-10-17

**Authors:** Evangelia Tarazi, Dimitra Statha, Christina Barda, Ioannis Sfiniadakis, Andreas Vitsos, Michail Christou Rallis

**Affiliations:** 1 Section of Pharmaceutical Technology, Department of Pharmacy, National and Kapodistrian University of Athens, Panepistimiopolis Zografou, 15784 Athens, Greecerallis@pharm.uoa.gr (M.C.R.); 2 Section of Pharmacognosy and Chemistry of Natural Products, Department of Pharmacy, National and Kapodistrian University of Athens, Panepistimiopolis Zografou, 15771 Athens, Greece; 3 Pathologoanatomic Laboratory, Athens Naval Hospital, 11521 Athens, Greece

**Keywords:** *Glycyrrhiza glabra* L., licorice, second degree burns

## Abstract

Second-degree burns are common dermal injuries requiring effective interventions to promote timely and complete skin regeneration. This study evaluated the wound-healing efficacy of topical hydrogels containing powdered licorice root (*Glycyrrhiza glabra* L.) extract at concentrations of 5%, 10%, and 20% *w*/*w* in a standardized murine model. Female SKH-hrHR2 hairless mice (*n* = 8 per group) were subjected to second-degree thermal burns, and treatment hydrogel formulations were applied once daily under occlusive dressings. Wound healing was assessed by planimetric area measurements, transepidermal water loss (TEWL), and histopathology. By Day 19, complete wound closure was achieved in 87.5% of animals in the 5% group, compared with 50.0% in the 10% group, 37.5% in the 20% group, and 25.0% in the sodium alginate control (Fisher’s exact test, *p* = 0.008). TEWL remained unchanged in the 5% group (baseline vs. Day 19: 8.4 ± 1.2 vs. 8.6 ± 1.3 g/m^2^/h; *p* > 0.05) but increased significantly in all other groups (e.g., sodium alginate: 8.2 ± 1.1 to 13.5 ± 2.0 g/m^2^/h; *p* = 0.0001). Histologically, the 5% formulation showed near-normal epidermal architecture and minimal inflammation (mean total score 2.0) compared with higher concentrations (6.0 for 10% and 7.3 for 20%) and sodium alginate (8.3). These findings demonstrate that a 5% *Glycyrrhiza glabra* hydrogel provides, among the concentrations studied here, the most favorable balance of wound closure, barrier restoration, and histological recovery, supporting its further development as a topical therapy for second-degree burns.

## 1. Introduction

Licorice (*Glycyrrhiza glabra* L.) has been widely used for centuries in traditional medicine systems across Asia, Europe, and the Middle East, valued for its broad therapeutic properties including anti-inflammatory, antimicrobial, and gastroprotective effects [1,2]. Beyond its well-known role in managing gastric ulcers and respiratory conditions, licorice root preparations have long been applied topically to soothe skin ailments and accelerate wound repair [3]. Recent research has confirmed that extracts of *G. glabra* exhibit potent antioxidant and anti-inflammatory activities, supporting their continued relevance as natural agents for promoting tissue regeneration [4].

The pharmacological profile of *Glycyrrhiza glabra* is largely attributed to its diverse set of bioactive constituents, including saponins (notably glycyrrhizin), flavonoids such as liquiritin, isoliquiritin, and glabridin, as well as various polysaccharides [4,5]. Glycyrrhizin, the major triterpenoid saponin, has been shown to exert strong anti-inflammatory [6] and suppress inducible nitric oxide synthase (iNOS) expression [7], thereby reducing oxidative and inflammatory damage [8]. Flavonoids such as isoliquiritin have demonstrated wound-healing potential through the promotion of angiogenesis and macrophage recruitment, which are essential processes in the inflammatory and proliferative phases of tissue repair [9]. Licoflavanone, another flavanone isolated from licorice leaves, was found to downregulate pro-inflammatory cytokines and inhibit the NF-κB/MAPK signaling pathway, highlighting its role as a modulator of inflammation-driven tissue injury [10]. In addition, glabridin has been reported to suppress matrix metalloproteinase expression through inhibition of NF-κB and AP-1 activity, further confirming the anti-inflammatory potential of licorice flavonoids [11]. Polysaccharide fractions also contribute to tissue protection by scavenging free radicals and enhancing collagen synthesis, thereby reducing inflammatory stress at the wound site [12]. Collectively, these compounds seem to act through complementary mechanisms that attenuate inflammatory responses while promoting tissue regeneration.

Second degree burns represent one of the most frequent skin’s inflammatory lesions seen in medical practice and are often difficult to manage effectively [13]. When such burns extend over wide surface areas or when treatment initiation is delayed, the likelihood of secondary infections increases substantially, posing a serious risk to patient outcomes [14]. Their management typically requires an integrated therapeutic strategy that may include topical and systemic antimicrobial agents, and in more severe cases, intravenous antibiotics or surgical interventions [15]. The healing of burn wounds follows a dynamic and tightly regulated process encompassing hemostasis, inflammation, proliferation, and remodeling, with each phase contributing critically to successful tissue regeneration [16]. Disturbances in this sequential cascade can result in delayed or incomplete repair, compromising both the structural and functional recovery of the skin [17]. These challenges underscore the urgent need for novel and effective therapeutic modalities to enhance the management of second-degree burns.

Hydrogel-based formulations offer significant advantages for the management of burn wounds, particularly in partial-thickness and deep burns. As described by Ringrose et al., hydrogels are hydrophilic polymers with high water content arranged in a three-dimensional network capable of absorbing exudate while maintaining a moist environment, which is critical to accelerate cell migration, proliferation, and epithelialization [18]. Their structure mimics the natural extracellular matrix (ECM), thereby promoting tissue integration, reducing fibrosis, and improving the elasticity and quality of the healed skin. Hydrogels also conform to irregular wound geometries more effectively than rigid dressings, enabling better coverage especially over complex surfaces [18]. In addition, functional hydrogels can be engineered with bioactive features—such as antimicrobial activity, anti-inflammatory modulation, controlled release of therapeutic agents, and stimuli-responsive behavior (e.g., responding to pH or temperature)—which together help address multiple challenges in burn wound healing: infection risk, excessive inflammation, delayed re-epithelialization, and scarring [18].

Sodium alginate, an anionic polysaccharide obtained from brown seaweed, is widely used as a wound dressing material due to its intrinsic biocompatibility, non-toxicity, and ability to form hydrogels under mild conditions that preserve the activity of incorporated bioactives [19]. Alginate hydrogels create a moist environment essential for cell migration and tissue regeneration, while their strong exudate-absorbing capacity helps prevent infection and maceration. Their porous, three-dimensional network supports oxygen exchange and can be easily further modified to achieve desirable mechanical stability and sustained release of therapeutic agents [20]. These features make sodium alginate an ideal natural polymeric matrix for delivering plant-derived bioactive extracts in advanced research of burn-wound treatments [21]. Moreover, as previously reported, polysaccharides such as alginate exhibit significant free-radical scavenging activity related to their structure–function characteristics [22]. Finally, previous studies have documented the compatibility of licorice extracts in formulations containing sodium alginate [23].

Given the above, it is not surprising that the therapeutic potential of licorice root in managing burn wounds is not a novel concept. In a recently conducted double-blind, randomized controlled trial, Zabihi and colleagues evaluated the efficacy of a hydroalcoholic *Glycyrrhiza glabra* extract embedded in a hydrogel for the treatment of second-degree burns in humans [24]. Their study involved approximately fifty patients who were randomly assigned to receive either the licorice extract-containing hydrogel or a placebo counterpart over a 15-day period. The licorice group experienced a significantly faster reduction in inflammation, redness, pain, and burning sensations—particularly notable between days 3 to 15, resulting in accelerated wound-healing compared to controls [24].

Building on these encouraging clinical findings, the present preclinical study was designed to explore dose–response relationships and to define the optimal extract concentration within a controlled experimental model, thereby providing quantitative evidence to complement and extend the clinical observations. To address this gap, we conducted what is, to our knowledge, the first systematic preclinical investigation of licorice hydroalcoholic extract incorporated into sodium alginate hydrogels, testing three defined concentrations (5%, 10%, and 20% *w*/*w*) in a standardized murine model of second-degree burns. By combining non-invasive biophysical measurements with detailed histopathological analysis, we aimed to define the concentration that achieves the most efficient wound closure and barrier restoration. This work therefore provides new and quantitative evidence on the optimal formulation parameters of licorice-based hydrogels and establishes a reproducible experimental framework that can guide subsequent mechanistic studies and clinical translation.

## 2. Results and Discussion

### 2.1. Results

#### 2.1.1. Systemic Observations

Throughout the experimental period, animals were monitored daily for general behavior (activity, grooming, and responsiveness), food and water intake, and local signs of irritation. No overt systemic or local adverse effects were observed in any treatment or control group. Body weight measurements remained stable and comparable across all groups, with no statistically significant intergroup differences (*p* > 0.05).

#### 2.1.2. Distribution of Complete Healing Across Treatment Groups

Contingency table analysis (Table 1) revealed significant differences in complete healing outcomes across the four treatment groups (Sodium Alginate, *Glycyrrhiza glabra* 5%, 10%, and 20% *w*/*w*). The Fisher’s exact test showed a statistically significant association between treatment group and healing status (*p* = 0.008), indicating that the probability of complete healing by Day 19 varied significantly depending on the treatment.

Post hoc analysis based on Pearson residuals identified that the Glycyrrhiza 5% group had a significantly higher number of healed animals than expected (residual = 2.500), while the Sodium Alginate group had fewer healed animals than expected (residual = −1.500), although this did not reach statistical significance. The Glycyrrhiza 10% and 20% groups showed residuals close to zero, suggesting outcomes were close to expected values under the null hypothesis of no association.

These findings suggest that the 5% Glycyrrhiza extract formulation significantly enhanced wound healing by Day 19 compared to the other treatments.

#### 2.1.3. Wound Area

**Figure 1:**
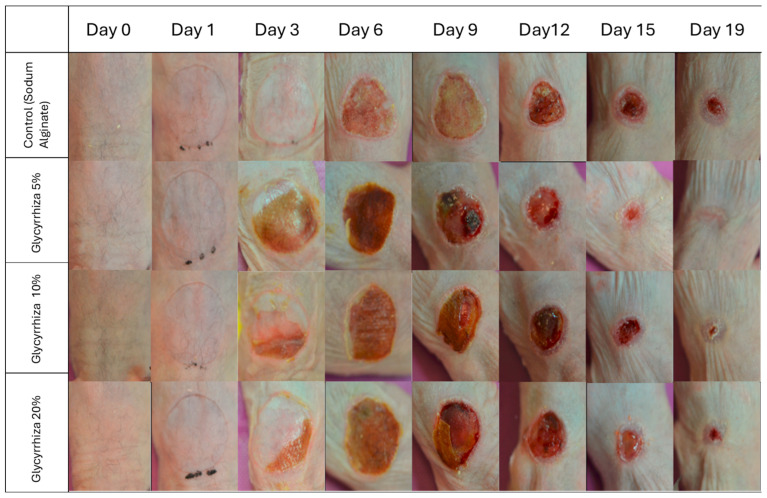
Representative macroscopic images of burn wounds during the healing process. Serial photographs are shown for each treatment group (rows: Sodium Alginate gel [control], Glycyrrhiza 5%, Glycyrrhiza 10%, and Glycyrrhiza 20%) across the study period (columns: Days 0, 1, 3, 6, 9, 12, 15, and 19). Images illustrate the progressive reduction in wound area and differences in the healing pattern between treatments. Images are provided for qualitative illustration; quantitative wound-area data and statistics are reported elsewhere.

The wound area evaluation is summarized in Table 2 and Figure 1.

Analysis of wound surface area revealed a significant time × group interaction (F(5.10, 47.60) = 3.46, *p* = 0.009, Greenhouse–Geisser corrected), suggesting that the temporal pattern of wound healing differed between treatments.

On the other hand, the between-subjects effect of group was not statistically significant (F(3, 28) = 1.84, *p* = 0.163), indicating that overall mean wound areas across the study period did not differ substantially among groups. Post hoc pairwise comparisons with Tukey’s correction confirmed the absence of statistically significant differences between treatment groups (all *p* > 0.17).

Complementary linear regression analysis supported these findings, showing a strong overall model fit (R^2^ = 0.809). Moreover, treatment group comparisons relative to sodium alginate were not statistically significant, although Glycyrrhiza 5% was only marginally non-significant, showing a trend toward faster wound healing (β = −0.082, SE = 0.044, *p* = 0.064).

#### 2.1.4. Results of the Histopathological Evaluation

**Figure 2:**
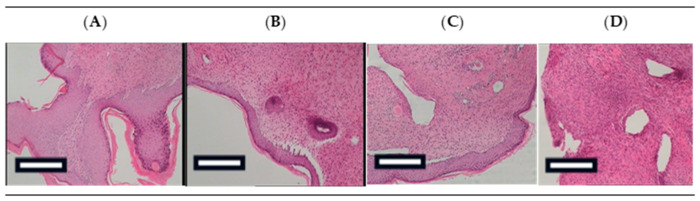
Representative histological sections of burn wounds on Day 19 (H&E stain, magnification 100×). (**A**) Sodium Alginate, (**B**) Glycyrrhiza 5%, (**C**) Glycyrrhiza 10%, and (**D**) Glycyrrhiza 20%. (Scale bar 100 μm).

Representative histological findings on Day 19 are summarized in Table 3 and illustrated in Figure 2.

In the sodium alginate group, epidermal hyperkeratosis was prominent, frequently accompanied by parakeratosis. Moderate dermal edema and a mild to moderate inflammatory infiltrate composed predominantly of polymorphonuclear cells and lymphocytes were also evident. The mean histopathological score was 8.3, indicating a less favorable healing profile.

In contrast, the Glycyrrhiza 5% group exhibited the most favorable histological picture. The epidermis resembled a nearly normal barrier structure, with minimal to absent inflammation, absence of hyperkeratosis or parakeratosis, and only limited edema. The mean score was 2.0, reflecting near-complete restoration of skin architecture.

The Glycyrrhiza 10% group displayed mild hyperkeratosis and a more evident inflammatory infiltrate compared with Glycyrrhiza 5%. Edema was relatively limited, and the overall histopathological outcome was better than that of sodium alginate but inferior to Glycyrrhiza 5%. The mean score for this group was 6.0.

Finally, the Glycyrrhiza 20% group showed extensive inflammation with abundant polymorphonuclear cells and mild hyperkeratosis, while edema remained limited. This group presented improved healing compared with sodium alginate, but the histological outcome was less favorable than that of Glycyrrhiza 5% and 10%. The mean score was 7.3.

Overall, these findings indicate that the Glycyrrhiza 5% treatment produced the most favorable histopathological healing response, characterized by minimal inflammation and near-normal epidermal morphology, whereas sodium alginate showed the poorest outcomes.

#### 2.1.5. Transepidermal Water Loss (TEWL)

**Figure 3:**
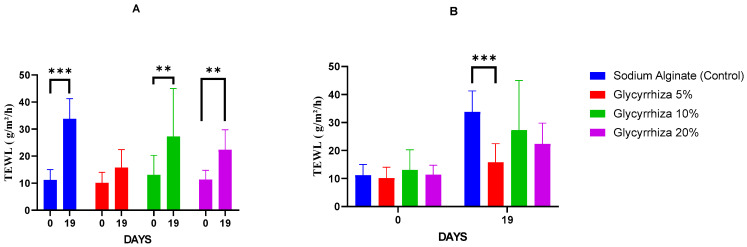
(**A**) Bar chart of within-group comparisons of transepidermal water loss (TEWL) at baseline (Day 0) and at the end of the experiment (Day 19). (**B**) Bar chart of between-group comparisons of TEWL at Day 0 and Day 19. Values are expressed as mean ± SD. Statistical significance is indicated with asterisks: *p* < 0.005 (**), *p* < 0.0001 (***) (Student’s *t*-tests, *n* = 8).

All results are highlighted in Figure 3A,B.

Baseline TEWL values (Day 0) did not differ significantly among the treatments. By Day 19, TEWL values had increased significantly compared to baseline in all treatments except Glycyrrhiza 5%. Specifically, significant increases were observed in sodium alginate (*p* = 0.0001), Glycyrrhiza 10% (*p* = 0.0078), and Glycyrrhiza 20% (*p* = 0.0017), consistent with the inflammatory response following burn injury. In contrast, the Glycyrrhiza 5% treatment did not show a statistically significant increase in TEWL between Day 0 and Day 19, indicating preservation of barrier function and effective wound repair in this group. Moreover, on Day 19, TEWL was significantly higher in the case of sodium alginate compared with Glycyrrhiza 5% (*p* = 0.0006).

### 2.2. Discussion

The present study investigated the wound-healing potential of hydrogels incorporating powdered *Glycyrrhiza glabra* root extract at concentrations of 5%, 10%, and 20%, using a murine model of second-degree burns. Our findings demonstrate that the 5% Glycyrrhiza extract formulation significantly enhanced wound healing, as evidenced by a higher proportion of completely healed animals by Day 19 (Table 1, Figure 1), a trend toward faster reduction in wound area (Table 2), improved skin barrier function (Figure 3), and markedly superior histological features compared to all other treatments (Figure 2, Table 3), including sodium alginate. These results are consistent with and expand upon previous reports of licorice’s anti-inflammatory and regenerative properties [9,10,24].

The contingency analysis (Table 1) revealed a statistically significant association between treatments and complete wound closure (*p* = 0.005), with the Glycyrrhiza 5% achieving the highest healing rates. Post hoc analysis of Pearson residuals confirmed this observation, identifying this treatment as significantly overperforming relative to the null hypothesis. In contrast, the 10% and 20% groups did not show similar outcomes, indicating a possible non-linear, concentration-dependent response.

This non-linear efficacy is further supported by the wound area measurements and regression analysis (Table 2). While the overall effect did not reach statistical significance (*p* = 0.163), the 5% Glycyrrhiza treatment demonstrated a trend toward more rapid wound contraction (β = −0.082, *p* = 0.064), suggesting potential clinical relevance. The lack of statistical significance across all treatment comparisons may be due to the relatively small sample size or variability in individual wound healing trajectories.

Interestingly, higher concentrations of Glycyrrhiza (10% and 20%) did not confer additional benefit and were associated with diminished healing performance. This phenomenon may be attributed to the potential cytostatic and antiproliferative effects of certain bioactive constituents, particularly Soliquiritigenin, as previously noted [25]. Indeed, Soliquiritigenin has been shown to act as a potent inhibitor of the NLRP3 inflammasome, suppressing its activation and delaying wound healing [25]. Another possible hypothesis involves the high content of flavonoids and other antioxidant compounds, which at elevated concentrations may paradoxically exert pro-oxidant activity [26], thereby promoting inflammatory processes rather than suppressing them, as also suggested by the histopathological findings showing enhanced oxidative stress at higher concentrations of Glycyrrhiza (Table 3). These observations highlight the importance of dose optimization in the development of herbal therapeutics.

Transepidermal water loss (TEWL) is a critical parameter for evaluating epidermal integrity during wound healing [27]. The clinical relevance of TEWL as a surrogate marker of barrier restoration is well established. As demonstrated in human burn patients, TEWL values progressively decline over time, reflecting scar maturation and functional recovery of the stratum corneum [28]. In our study, TEWL values remained statistically unchanged from baseline in the Glycyrrhiza 5% group, unlike the sodium alginate and higher licorice concentrations, where TEWL increased significantly by Day 19 (Figure 3). This preservation of barrier function suggests more effective and complete re-epithelialization in the 5% group and supports the hypothesis that licorice extract can promote functional skin regeneration, not merely morphological repair.

Histological analysis confirmed the superior regenerative capacity of the 5% Glycyrrhiza formulation (Figure 2, Table 3). The skin samples from this group exhibited near-normal epidermal architecture with minimal inflammation, no parakeratosis or hyperkeratosis, and the lowest total histopathological score (2.0) (Table 3). In contrast, sodium alginate-treated wounds showed persistent hyperkeratosis, moderate inflammation, and the poorest overall histological profile.

These results align with existing literature on the wound-modulating effects of glycyrrhizin and licorice-derived flavonoids, which act through downregulation of NF-κB signaling and suppression of pro-inflammatory cytokines [2,10]. Flavonoids such as isoliquiritin have also been shown to enhance angiogenesis and macrophage recruitment, key events in the proliferative phase of wound healing [9].

The evidence presented here suggests that a 5% concentration of *Glycyrrhiza glabra* in hydrogel formulations offers an optimal balance between efficacy and tolerability for the treatment of second-degree burns. Its ability to accelerate wound closure, preserve barrier function, and promote histological regeneration supports further exploration in preclinical and clinical contexts.

Despite its importance, this study has some limitations. Most notably, it does not provide mechanistic insights into how the licorice extract promotes wound healing. Key molecular and cellular pathways—such as inflammatory cytokine expression, oxidative stress regulation, immune cell infiltration, angiogenesis, fibroblast activation, collagen remodeling, and re-epithelialization—were not evaluated. As this work was conceived as a first-step preclinical dose–response study, mechanistic markers were beyond its initial scope. Furthermore, the modest sample size (*n* = 8 per group) may limit statistical power; however, this was mitigated by applying Fisher’s exact test and by focusing the study on comparative rather than population-level estimates. Future investigations should include targeted molecular analyses in larger samples to clarify the extract’s mode of action and support causal inferences.

Another limitation often highlighted in herbal research is the lack of chemical characterization and standardization of the extract. In our case, this was not pursued, as *Glycyrrhiza glabra* is a well-known medicinal plant that has been extensively analyzed in literature, with its key bioactive constituents thoroughly described. Furthermore, the raw material for crude powdered-root hydroalcoholic preparation used in this study was obtained from an established supplier of raw materials for the pharmaceutical and cosmetic industries, accompanied by specifications and quality controls. Given the plant’s well-documented phytochemical profile, further analysis was not deemed necessary for the purposes of this work. An additional limitation of this study is that only a hydroalcoholic extract of *Glycyrrhiza glabra* was evaluated; other extraction methods (e.g., aqueous or supercritical) may produce different phytochemical profiles and biological activities, which were not compared here. Future studies may incorporate additional standardization steps, lower doses than 5% and targeted fractionation approaches to link specific marker compounds with mechanistic outcomes. Nevertheless, this study, as a first step, provides important directions to guide the design of subsequent investigations.

## 3. Conclusions

This study demonstrated that topical application of *Glycyrrhiza glabra* extract at a concentration of 5% significantly enhances the healing of second-degree burns in a murine model. Among all tested formulations, the 5% hydrogel exhibited the most favorable outcomes across primary and secondary endpoints, including a higher rate of complete wound closure, accelerated reduction in wound area, preservation of skin barrier function, and near-normal histological architecture by the study’s conclusion.

Higher concentrations (10% and 20%) did not yield comparable benefits and were associated with less favorable healing profiles, highlighting the importance of dose optimization in the development of phytotherapeutic agents. The results suggest that the 5% Glycyrrhiza formulation may represent a promising candidate for further investigation in preclinical and clinical settings, offering a natural, accessible, and effective option for the topical management of second-degree burn wounds.

Future studies should focus on standardizing the bioactive constituents of licorice extracts, elucidating the underlying molecular mechanisms, and validating these findings in human clinical trials.

## 4. Materials and Methods

### 4.1. Preparation of Hydrogels

Powdered roots of *Glycyrrhiza glabra* L. were purchased (Syndesmos S.A., Athens, Greece). A hydroalcoholic extract was prepared using a solvent mixture of ethanol and water (70:30 *v*/*v*). Briefly, dried powdered root material was fully covered with the solvent mixture. After 24 h, the first filtrate was collected, and fresh solvent was added again. This extraction step was repeated three times to ensure recovery of most extractable constituents. The combined filtrates were concentrated under reduced pressure using a rotary evaporator (IKA^®^-Werke GmbH & Co. KG, Staufen, Germany) and subsequently lyophilized (Alpha 1–5 Freeze dryer, Martin Christ Gefriertrocknungsanlagen GmbH, Osterode am Harz, Germany), yielding a dry hydroalcoholic extract in powdered form with an approximate yield of 15% *w*/*w*.

Hydrogels were formulated using sodium alginate (Syndesmos S.A., Athens, Greece) as a viscosity enhancer (5% *w*/*w*) and stabilizing agent. Three formulations were prepared containing 5%, 10%, and 20% *w*/*w* licorice extract, respectively. For the 5% *w*/*w* formulation, 5 g of powdered hydroalcoholic licorice extract was dispersed in 90 g sterile water, followed by gradual addition of 5 g sodium alginate under continuous stirring until a homogeneous gel was obtained. The 10% *w*/*w* formulation was prepared by mixing 10 g extract with 85 g sterile water and 5 g sodium alginate, while the 20% *w*/*w* formulation contained 20 g extract, 75 g sterile water, and 5 g sodium alginate. Final gels were stored in sealed plastic containers at cooled conditions (4–8 °C) until use.

### 4.2. Experimental Animals

The study was conducted under the approved experimental license (863612/11-07-2025), issued by the Veterinary Directorate of the Region of Attica, Greece. In accordance with the European Council Directive 2010/63/EU on the protection of animals used for scientific purposes, all experimental procedures were performed at the accredited Small Laboratory Animal Facility of the Division of Pharmaceutical Technology, Department of Pharmacy, National and Kapodistrian University of Athens (NKUA), with registration code EL 25 BIOexp 07. All procedures were performed in accordance with ARRIVE guidelines [29].

A total of thirty-two female hairless mice (SKH-hr2 strain), aged 2–5 months, were used. The animals originated from the breeding and experimentation unit of the Division of Pharmaceutical Technology, Department of Pharmacy, NKUA (Athens, Greece). Mice were housed in groups of eight in standard cages, under controlled environmental conditions of temperature, humidity, air renewal, and a 12 h light/dark cycle. Food and water were provided ad libitum, and hygiene was carefully maintained throughout the study.

Animals were randomly assigned to four experimental treatments (*n* = 8 per group) using age stratification [30], as follows:

1. Treatment 1 (Control): Sodium alginate 5% hydrogel (vehicle control)
2. Treatment 2: 5% *Glycyrrhiza glabra* hydroalcoholic extract hydrogel
3. Treatment 3: 10% *Glycyrrhiza glabra* hydroalcoholic extract hydrogel
4. Treatment 4: 20% *Glycyrrhiza glabra* hydroalcoholic extract hydrogel

The required sample size was estimated using the software G*Power version 3.1.9.7 (Heinrich Heine University, Düsseldorf, Germany). A repeated measures ANOVA with a between–within interaction design was selected to reflect the experimental conditions involving four treatment modalities and five repeated measurements of wound area over time. The analysis was based on an assumed medium effect size (f = 0.25), with α = 0.05, power (1 − β) = 0.95, correlation among repeated measures = 0.5, and nonsphericity correction ε = 1. Based on these parameters, the minimum total sample size required was 32 animals, corresponding to 8 mice per group. This sample size was deemed adequate to detect statistically significant differences in wound healing trajectories among treatment groups with sufficient power.

### 4.3. Burn Wound Infliction and Maintenance

Burn injuries were inflicted following a prior standardized and reproducible protocol [31]. Mice were anesthetized via intraperitoneal injection of 100 mg/kg ketamine (Ketamidor, 100 mg/mL, Richter Pharma AG, Wels, Austria) and 7 mg/kg xylazine (Xylapan, 20 mg/mL, Vetoquinol SA, Lure Cedex, France). Each animal was positioned ventrally over a narrow sponge to stabilize the body and isolate the forelimbs from the hindlimbs, ensuring uniform access to the dorsal skin.

Burn wounds were created on the upper back, precisely 2 cm below the base of the ears, using a circular metal stamp with a surface area of 2 cm^2^ [31]. The stamp was preheated in a water bath at 69 ± 2 °C for 3 min, then quickly blotted with cloth to remove surface water. It was then applied with gentle pressure to the stretched dorsal skin for exactly 10 s. Between applications, the stamp was returned to the water bath for at least 3 min to reattain the desired temperature. The procedure was performed sequentially across all groups, ensuring consistent timing and thermal conditions.

Immediately following burn induction, the wound was photographed, the respective gel formulation was topically applied, and the area was covered with a dressing consisting of Fixomull Stretch self-adhesive gauze (BSN Medical, Hamburg, Germany) layered with Medicomp non-woven sterile gauze (Hartmann, Hamburg, Germany). The adhesive was cut into a hexagonal shape to avoid impairing the mobility of the mice.

To manage pain, paracetamol (Vianex, Athens, Greece) was administered in the drinking water at a concentration of 1.0 mg/mL for three consecutive days post-burn. Treatments were applied once daily until the completion of the experiment. The study was terminated when at least 80% of the animals within any treatment group had achieved complete wound closure, indicating sufficient re-epithelialization and ensuring a humane endpoint consistent with the 3Rs principle.

A standardized and consistent wound care protocol was applied across all experimental groups throughout the study period. Once daily, the existing dressing was gently removed using forceps after being moistened with sterile injectable water for approximately five minutes to avoid mechanical irritation. The wound area was then carefully cleansed by dabbing with cotton soaked in injectable water. Subsequently, the designated hydrogel formulation was applied directly to the burn site using a sterile spatula. Each application delivered a standardized dose of approximately 40 mg/cm^2^. Finally, the treated area was covered with sterile non-adherent gauze and secured with adhesive tape as described previously.

### 4.4. Burn Wound Healing Assessment

#### 4.4.1. Macroscopic Evaluation and Wound Area Analysis

Macroscopic evaluation of the wounds was performed daily throughout the study. Photographic documentation was conducted using a Nikon D5100 digital camera equipped with an AF-S Micro Nikkor 60 mm f/2.8 G ED lens (Nikon, Tokyo, Japan). The camera was positioned at a fixed vertical distance of 20 cm from the dorsal surface of each mouse to ensure standardization. Photographs were taken on the first day and every two days thereafter.

#### 4.4.2. Evaluation of Transepidermal Water Loss (TEWL)

Transepidermal water loss (TEWL), a key biophysical parameter of skin barrier function, was assessed using a non-invasive method as described elsewhere [28]. Measurements were obtained at two time points: baseline (prior to burn induction) and at the end of the treatment period. TEWL was expressed in grams per square meter per hour (g/m^2^/h) and measured using the Tewameter^®^ TM 300 (Courage + Khazaka Electronic GmbH, Cologne, Germany).

To ensure accuracy and consistency, all assessments were performed under controlled environmental conditions. Prior to each measurement, the skin surface was gently cleansed with sterile gauze to eliminate contaminants or residual substances that could interfere with device readings.

#### 4.4.3. Histopathological Evaluation

At the termination of the experiment, all animals were euthanized via cervical dislocation under isoflurane anesthesia [32]. Full-thickness skin samples were excised from the center of the burn wound area and immediately fixed in 37% formaldehyde. The specimens were subsequently processed, embedded in paraffin, and sectioned to a thickness of 5 µm.

Histological staining was performed using hematoxylin and eosin (H&E). The prepared slides were examined under 100× magnification using a multi-head light microscope (Nikon Eclipse 50, Nikon Corp., Tokyo, Japan). Assessment was conducted in a blinded manner, with scorers unaware of the treatment allocation of each sample.

The histopathological evaluation focused on seven parameters: the degree of inflammatory cell infiltration, the presence and extent of edema, hyperkeratosis, ulceration, necrosis, parakeratosis, and overall wound thickness. Scoring was performed according to a predefined semi-quantitative scale, as detailed in Table 4. For each parameter, the scores assigned to animals within a group were averaged to obtain a mean value. These mean values for the seven parameters were then summed to calculate the overall histology score for each group.

### 4.5. Statistical Analysis

Statistical analyses were performed using the Jamovi Cloud 2.7.6.platform (jamovi.org, accessed on 15 October 2025) [33], and all graphical representations were created with GraphPad Prism (version 8.4.3, GraphPad Software, San Diego, CA, USA). A significance level of *p* < 0.05 was set for all comparisons. To assess the association between treatment group and complete wound healing by Day 19, a contingency table analysis was conducted using Fisher’s exact test suitable for small samples. Pearson residuals were used for post hoc evaluation to identify specific cells that significantly contributed to the overall chi-square result.

For the assessment of wound area over time, a repeated-measures ANOVA was applied, with the Greenhouse-Geisser correction used in cases of sphericity violation. A linear regression analysis was also performed to evaluate the effect of time (day) and treatment group on wound area reduction. TEWL data were analyzed using P *t*-tests for within-group comparisons and independent-sample *t*-tests for between-group comparisons.

## Figures and Tables

**Table 1:** Complete wound healing by Day 19 across treatment groups: contingency analysis, overall significance tests, and post hoc residuals. Significance set at *p* < 0.05. (**A**) Contingency table showing the distribution of animals with incomplete healing (event = 0) and complete healing (event = 1) by Day 19 in each treatment group. (**B**) Results of χ^2^ test and Fisher’s exact test assessing the overall association between treatment group and healing outcome. (**C**) Post hoc analysis using Pearson residuals, indicating deviations from expected frequencies under the null hypothesis. Residuals greater than +1.96 or less than −1.96 highlight cells that contribute significantly to the overall chi-square results (*n* = 8).

(A)				(B)				(C)		
Contingency Tables				χ^2^ Tests				Post Hoc Test (Pearson Residuals)		
	Event				Value	df	*p*		Event	
Treatment	0	1	Total					Treatment	0	1
Sodium Alginate	8	0	8	χ^2^	12.8	3	0.005	Sod Alginate	0.938	−1.500
Glycyrrhiza 5%	2	6	8	Fisher’s exact test			0.008	Glycyrrhiza 5%	−1.564	2.500
Glycyrrhiza 10%	6	2	8	N	32			Glycyrrhiza 10%	0.104	−0.167
Glycyrrhiza 20%	7	1	8					Glycyrrhiza 20%	0.521	−0.833
Total	23	9	32							

**Table 2:** Linear regression model coefficients for wound area reduction. The model included time (day) and treatment group as predictors, with Sodium Alginate as the reference category. A significant negative association was observed between day and wound area (*p* < 0.001), indicating progressive reduction in wound size over time. None of the group comparisons reached statistical significance, although Glycyrrhiza 5% demonstrated a trend toward faster wound contraction relative to Sodium Alginate (*p* = 0.064). (*n =* 8).

Model Coefficients—Wound Area				
Predictor	Estimate	SE	t	*p*
Intercept ^a^	1.69185	0.05220	32.414	<0.001
Group:				
Glycyrrhiza 5%–Sodium Alginate	−0.08212	0.04404	−1.865	0.064
Glycyrrhiza 10%–Sodium Alginate	−0.00912	0.04404	−0.207	0.836
Glycyrrhiza 20%–Sodium Alginate	−0.01655	0.04404	−0.376	0.708
Time	−0.08771	0.00343	−25.544	<0.001

^a^ Represents reference level.

**Table 3:** Mean histopathological scores of burn wounds on Day 19. Values represent mean scores for each parameter (inflammation, edema, hyperkeratosis, and lesion thickness) per treatment group. The Total Score corresponds to the sum of individual parameters and reflects the overall histopathological severity (*n* = 3).

Treatment	Inflammation	Oedema	Hyperkeratosis	Wound Thickness	Total Score
Control (Sodum Alginate)	1.67 ± 0.58	2 ± 0	2.67 ± 0.58	2 ± 0	8.33 ± 0.58
Glycyrrhiza 5%	0.67 ± 0.58	1 ± 0	0.33 ± 0.58	0 ± 0	2 ± 1
Glycyrrhiza 10%	2 ± 0	1 ± 0	1.67 ± 0.58	1.33 ± 0.58	6 ± 1
Glycyrrhiza 20%	2.67 ± 0.58	1 ± 0	2 ± 0	1.67 ± 0.58	7.33 ± 0.58

**Table 4:** Semi-quantitative histopathological scoring criteria used for the evaluation of burn wound tissue samples. Each parameter was graded on a scale from 0 to 3 (0 = absence, 1 = mild, 2 = moderate, 3 = heavy presence). For each group, scores for each parameter were averaged across animals, and these mean values were then summed to obtain the overall histology score. Evaluation was performed in a blinded fashion under 100× magnification.

	Absence	Mild	Moderate	Heavy
Inflammation	0	1	2	3
Oedema	0	1	2	3
Hyperkeratosis	0	1	2	3
Wound thickness	0	1	2	3

## Data Availability

The data that support the findings of this study are available from the corresponding author upon reasonable request.
